# Adenine as Epoxy Resin Hardener for Sustainable Composites Production with Recycled Carbon Fibers and Cellulosic Fibers

**DOI:** 10.3390/polym12123054

**Published:** 2020-12-20

**Authors:** Stefano Merighi, Laura Mazzocchetti, Tiziana Benelli, Loris Giorgini

**Affiliations:** 1 Department of Industrial Chemistry “Toso Montanari” and INSTM UdR-Bologna, University of Bologna, Viale Risorgimento 4, 40136 Bologna, Italy; stefano.merighi2@unibo.it (S.M.); laura.mazzocchetti@unibo.it (L.M.); tiziana.benelli@unibo.it (T.B.); 2 Interdepartmental Center for Industrial Research on Advanced Applications in Mechanical Engineering and Materials Technology, CIRI-MAM, University of Bologna, Viale Risorgimento 2, 40136 Bologna, Italy

**Keywords:** adenine, epoxy resins, biobased hardener, recycled carbon fibers, jute fibers, flax fibers, FRP, composite, sustainability

## Abstract

In this work, Adenine is proposed, for the first time, as a cross-linker for epoxy resins. Adenine is an amino-substituted purine with heterocyclic aromatic structure showing both proton donors, and hydrogen bonding ability. DSC studies show that adenine is able to positively cross-link a biobased DGEBA-like commercial epoxy precursor with good thermal performance and a reaction mechanism based on a ^1^H NMR investigation has been proposed. The use of such a formulation to produce composite with recycled short carbon fibers (and virgin ones for the sake of comparison), as well as jute and linen natural fibers as sustainable reinforcements, leads to materials with high compaction and fiber content. The curing cycle was optimized for both carbon fiber and natural fiber reinforced materials, with the aim to achieve the better final properties. All composites produced display good thermal and mechanical properties with glass transition in the range of HT resins (*T*_g_ > 150 °C, E’ =26 GPa) for the carbon fiber-based composites. The natural fiber-based composites display slightly lower performance that is nonetheless good compared with standard composite performance (*T*_g_ about 115–120 °C, E’ = 7–9 GPa). The present results thus pave the way to the application of adenine as hardener system for composites production.

## 1. Introduction

Fiber reinforced polymers (FRPs), well renowned for their excellent specific mechanical properties, are more and more applied in lightweight requiring field, with the aim of reducing fuel consumption and increase durability. While their premium properties are undoubtfully appreciated when trying to increase sustainability of, among the other, vehicles, wind blades or airplanes; FRPs, however, lack an intrinsic material sustainability that hampers a full green revolution when they are involved [1]. Indeed, beside their significant cost, they are fully fossil-based materials and, in particular, when carbon fibers (CFs) are used as reinforcement, the processing is extremely highly energy-requiring, making CFs, and carbon fiber reinforced polymers (CFRPs), in general, far from sustainable [2,3]. Additionally, their end-of-life disposal poses some serious issue, since the composite structure, which is based on an intimate contact of different phases, is practically impossible to easily reverse [4,5]. As a result, in the last few years, there has been an increasingly renewed interest in sustainable composite materials that are environmentally safe and have high performance. Indeed, the need to become sustainable in practically all fields is a recent claim that is receiving growing attention; in recent years, this aim also extends to finding next generation feedstocks from non-food competitive resources, such as waste or low added-value supplies. It is, thus, clear that extending this concept to composites means acting both on the matrix and on the reinforcement simultaneously [6]. In this context, bio-based resins [7] are now a commercially developed and expanding product, and are often only partially derived from biological—mostly vegetable—feedstocks, such as catechin [8,9], cardanol [10] and lignin [11,12]. The biobased fraction in the resin, however, often refers mainly to the epoxy prepolymer component, while the hardener sustainability [13] is mostly disregarded when high performances are sought [14], with fossil-based diamine curing agents, such as 4,4′-methylene biscyclohexanamine (PACM) [15,16] and diethyl toluene diamine (EPIKURE W) [17,18], that are commonly used to obtain high thermomechanical properties. Indeed, the structure and functionality of hardener is one of the key factors in the definition and achievement of the final properties of the thermoset, and the prospect of access to a library of sustainable cross-linking systems is one that is highly appealing [19,20,21,22,23]. 

While a wealth of literature is devoted to the production of resins from renewable resources [24,25,26,27], the easiest approach to sustainability in fiber reinforced composites is the switch to natural fibers [28] and, as has been conducted only recently, in the attempt to recycle composites to reuse, at the very least, the reinforcing fraction [29,30,31,32]. 

Natural fibers (NF) [33,34,35], which are well renowned for their textile use, can also be used for composite production: in this field, however, the cheaper and lower quality bast fibers are used instead of fibers obtained from seeds, such as cotton [36]. Bast fibers are characterized by a lower cellulose content and the presence of lignin that is missing in seed fibers [36] and is considered responsible for increasing equilibrium moisture contents (EMC), owing to lignin ability to absorb water and deform [27]. Among bast fibers, flax fibers appear as some of the most promising candidates. Flax, or linseed, is grown for its seeds’ fibers, which are highly cellulosic and are used to produce linen textile, while flax fibers are extracted from the bast or skin of the stem of the plant. They are characterized by an average low lignin content and high cellulose fraction that guarantees good mechanical properties and strength to the fiber: however, they are still costly, ranging in between glass and carbon fibers in price, and they are, as with all natural fibers, short filaments that need to be twisted into yarns, thus reducing their mechanical performance. A cheaper alternative to flax is jute fiber, one of the most affordable natural fibers, second only to cotton in the amount produced and variety of uses; it has a similar cellulosic content but a higher lignin fraction, making them more hygroscopic and potentially difficult to be processed. The application of these fibers in different contexts has been widely reported [37,38,39]. While their average performance can be acceptable for standard applications, which simply pursues light-weighting as goal, when a composite needs to withstand a relevant mechanical performance, such as in primary load bearing structures, the use of natural fibers is still not enough to match the target [40,41,42]. In the latter case, the performance of glass or carbon fibers is required and a viable approach for making the composite more sustainable is the use of recycled fibers [4,43]. A particularly positive approach for recycling CF from CFRP (end-of-life wastes or production scraps) is the pyrolysis, a thermochemical process able to reach already industrial application, even if in a still limited dimension. Pyrolysis is indeed a flexible and reliable process able to treat different complex substrates and recover the reinforcing fraction, irrespective of its shape a material [4,30,32,44]. This technique is not suitable for recovering natural fibers, but when optimized is able to give back both glass and carbon fibers [30,32,44], with the latter reinforcement that is proved to keep 85–90% of the original CF mechanical properties [4,43]. 

**Scheme 1:**
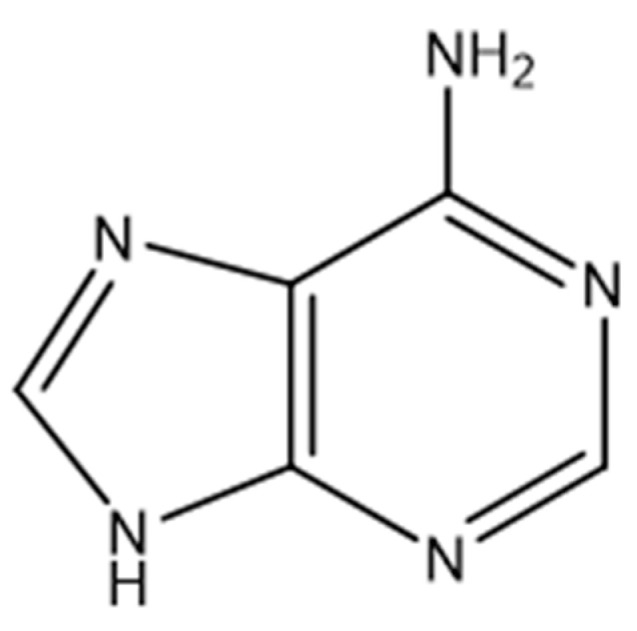
Structure of Adenine (A).

In the present work, the authors tackle the issue of composite sustainability under different sequential pathways. First of all, the production of epoxy resin formulations making use of adenine as a biobased cross-linker for partially biobased epoxy precursor is addressed. Adenine (Scheme 1) is an amino-substituted purine known to be an important part of DNA. This molecule has gained a lot of attention due to its marked bio-activity and unique bio-compatibility. Several studies have also shown that Adenine can easily be obtained on an industrial scale by fermentation processes [45]. Adenine, however, due to its particular heterocyclic aromatic structure showing both proton donors (–NH–, =CH–), acceptors (=N–, C=O) and hydrogen bonding ability, shows potential in terms of design of high-performance thermosets [46], with a targeted high *T*_g_ to be used in high temperature (HT) applications. As previously discussed, biobased hardeners leading to resins for HT application are not common and they might gain a high interest in the field. Adenine can be produced from DNA degradation and specifically obtained by fermentation through the production of adenosine [47,48]. Fermentation occurs mainly on an industrial scale through microbial fermentation, with yields up to 16.4 g/L [49]. With the aim of assessing Adenine viability as a hardener for epoxy resin production, a commercial bio-based epoxy precursor was chosen—SUPER SAP^®^ CLR (Entropy Resin)—that in commercial formulations with the non-biobased suggested hardeners is reported to reach reasonable mechanical properties (*T*_g_ about 65–70 °C, and both Tensile and Flexural Moduli about 3 GPa, depending on the specific hardener used) [50]. The reaction occurrence was evaluated via dynamic scanning calorimetry (DSC) measurements and an attempt at establishing a reactivity pattern was analyzed via ^1^H NMR spectroscopy in different condition, using a model epoxy compound that should react similarly to the epoxy precursor, but without cross-linking.

The main aim of this paper is, however, twofold, and the work will thus continue exploiting this newly formulated resin to produce fiber reinforced composites (FRCs). Indeed, not many works analyze the full composite production stemming from the resin reactivity to its final ability to obtain viable composites. In the present case, thus, sustainable reinforcements, such as natural fibers (jute and flax fibers) and recycled carbon fibers (rCFs) obtained via pyro-gasification process was applied. In the latter case for the sake of comparison, analogous virgin fibers (vCFs) are also used as reinforcement, in order to highlight the differences with the recycled ones. The resin formulations and the obtained composite products have been tested via thermogravimetric analysis (TGA), dynamic scanning calorimetry (DSC) and dynamic mechanical analysis (DMA).

## 2. Materials and Methods 

### 2.1. Materials

SUPER SAP^®^ CLR (hereafter CLR) resin was purchased from Entropy Resins (Gougeon Brothers Inc., Bay City, MI, USA) and used as received. Adenine (99% pure) was purchased from Alfa Aesar (Thermo Fisher GmbH, Kandel, Germany) and used as received. Glycidyl 2-Methylphenyl Ether (90%) (G2MPE) was purchased from Sigma Aldrich (Merck KGaA, Darmstadt, Germany). Virgin chopped carbon fibers (vCF) (25 mm) were obtained cutting down Unidirectional Fabric UC 301 based on Toray T700S 12K dry fibers (Toray Industries Inc., Tokyo, Japan). Recycled chopped carbon fibers (rCF) (25 mm) were obtained by pyrolysis (500 °C for 150 min) of Toray T700S 12K-based carbon fiber composites post-treated at 500 °C for 60 min under oxidizing atmosphere in collaboration with Curti SpA (Curti Spa, Castel Bolognese, Italy) [30,43]. Both virgin and recycled carbon fibers were used without any other treatment. Flax and jute fibers (FF and JF), kindly supplied from Assen University (Netherlands), were manually chopped at a length of 25 mm and used after drying overnight at 110 °C.

#### 2.1.1. Resins Formulations Preparation

Different amounts of Adenine (1.7, 3.5, and 6.0 mmol) were added to SUPER SAP^®^ CLR resin (29.4 mmol, Table 1). The obtained formulations (named CA1, CA2 and CA3, respectively) were mechanically stirred at 300 rpm and heated at 75 °C until complete homogenization of the hardener with the resin. Then, they were cooled down to room temperature and used for the preparation of TGA and DSC samples. 

#### 2.1.2. Chopped Carbon Fiber Reinforced Composites Production

Virgin (vCF) or recycled carbon (rCF) fibers of an average 25 mm length (18 g) were added to the pre-mixed CA2 formulation (15 g) in a fiber/matrix 55/45 wt% ratio at 7 5°C until complete uptake of the resin by the fibers.

Then the resin/carbon fiber mixtures (33 g) were poured into a 60 × 60 × 10 mm^3^ steel mold and hot-press cured at 50 bar pressure according to the following procedure: the mold was inserted in the press with the plates already heated at 140 °C, where it was kept in isotherm for 45 min with an applied pressure of 1 bar. After the isothermal step, the temperature was raised to 180 °C and the applied pressure was increased up to 50 bar. The sample was kept in this isothermal condition for 200 min. Finally, before opening the hot-press plates, the mold was cooled down to RT. The obtained composite panels, named CA-vCF and CA-rCF, were cut into 100 × 15 mm^2^ strips in order to perform thermal and mechanical characterization.

#### 2.1.3. Chopped Natural Fiber Reinforced Composites Production

Dried flax (FF) or jute (JF) fibers (25 mm length, 10 g) were added and carefully hand-stirred with the pre-mixed CA2 system in a 50:50 weight ratio at 75 °C until complete uptake of the resin by the fibers. The mixtures (20 g) were poured into a steel mold with a 60 × 60 × 10 mm^3^ cavity, which was closed with a counter-mold designed to allow the resin excess to come out from the edges. Hence, the system was placed in a hot-press and cured at 50 bar pressure according to the following procedure: the mold was inserted in the press with the plates already heated at 160 °C, where it was kept in isotherm for 45 min with an applied pressure of 1 bar. After the isothermal step, the temperature was raised to 190 °C and the applied pressure was increased up to 50 bar. The sample was kept in this isothermal condition for 200 min. Finally, before opening the hot-press plates, the mold was cooled down to RT. Each panel was post-cured at 180 °C for 120 min in order to complete the cross-linking reaction of the resin. The obtained panels, named CA-FF and CA-JF, were cut into 100 × 15 mm^2^ strips in order to perform thermal and mechanical characterization.

### 2.2. Methods

The composite formulations were cured in a hot-press (DAN12T4CPT model, produced by Monti Sistemi, Bologna, Italy) equipped with water cooling plates. ^1^H NMR spectra were obtained on 5–10% *w*/*v* CDCl_3_ (at room temperature) or DMSO (at 90 °C) solutions, using a Varian MercuryPlus VX 400 (^1^H, 399.9; C, 100.6 MHz) spectrometer (Agilent Technologies, Inc., Santa Clara, CA, USA). Chemical shifts are given in ppm from tetramethylsilane (TMS) as the internal reference. The thermal behavior of the reacting mixtures was evaluated by differential scanning calorimetry (DSC, Q2000 TA Instruments Inc., New Castle, DE, USA) carrying out the measurements in dynamic and isothermal mode. Dynamic DSC analyses were performed under nitrogen flow, heating the sample at a heating rate of 1 °C/min from −50 to 280 °C. After the first heating, the sample was cooled down to 0 °C and heated again with a heating rate of 20 °C/min from 0 to 260 °C for *T*_g_ evaluation. Isothermal DSC analyses were performed at 160, 180, and 200 °C, introducing the sample in the pre-heated furnace. After the isothermal step, the sample was cooled down to 0 °C and then heated again with a heating rate of 20 °C/min from 0 to 260 °C for *T*_g_ evaluation. Thermogravimetric analysis (TGA) was carried out on a TA Instruments SDT Q600 (TA Instruments Inc., New Castle, DE, USA). Carbon fiber-based composites were analyzed according to the following program: a first heating step in inert atmosphere (nitrogen flow rate 100 mL/min) heating from RT to 500 °C at 20 °C/min is followed by 5′ isotherm before cooling down to 300 °C at −20 °C/min with addition 5′ isotherm at 300 °C. Then atmosphere is switched to oxidizing atmosphere (air, flow rate 100mL/min) and heating up to 500 °C at 10 °C/min followed by 20′ isotherm. Natural fiber-based composites were simply heated in inert atmosphere (nitrogen flow, rate 100 mL/min) at 20 °C/min from RT to 500 °C. Dynamic mechanical analysis (DMA) was performed with Netzsch 242 E Artemis analyzer (NETZSCH Holding, Selb, Germany), in three point bending configuration with a 20 mm sample holder. The following conditions, in mixed control, were used during the analysis: heating ramp at 3 °C/min from RT up to 250 °C, frequency 1 Hz, maximum amplitude ±10 µm, maximum dynamic force 10 N, additional static force 0.5 N and proportional factor (Fstat/Fdin) = 1.1. Samples for DMA are about 50 × 10 × 2.5 mm^3^ (the exact dimensions were measured for each coupon).

## 3. Results and Discussion

With the aim of testing the cross-linking ability of Adenine acting as hardener system, an attempt to react it with a partially biobased epoxy precursor has been carried out. The resin of choice, SUPER SAP^®^ CLR (Entropy Resins) (hereafter called simply CLR), is said to be 28.6% biobased, as certified by US Department of Agriculture (USDA) [51]. A ^1^H and ^13^C NMR investigation of the commercial resin (see Figure S1 in Supplementary Materials) shows that it is substantially a Diglycidyl Ether of Bisphenol A (DGEBA) derivative. 

While the CLR technical datasheet [50] supplied by the producer states that the resin cured at room temperature (RT) in the presence of their different commercial hardening counterpart can reach glass transition temperature (*T*_g_) slightly above RT (61–66 °C), previous studies [52] proved that this type of epoxy precursor is also able to react with L-Tryptophan reaching *T*_g_ as high as 100 °C. Indeed, the presence of a nitrogenated heterocyclic system, with a lateral aliphatic chain, seems to promote the reaction attaining higher stiffness of the thermoset. The use of Adenine (Scheme 1), being a hetero-aromatic structure bearing different amine functional groups onto the stiff aromatic structure, without any pendant moiety as L-Tryptophan, is expected to perform even better. While technical datasheet states an epoxy/precursor weight ratio of about 100/50, the attempt at reaching such a high hardener fraction in the Adenine formulations highlights a solubility threshold: a homogeneous mixture, even when the components are heated at 75 °C, can be obtained up to an Adenine content of 7.5 wt%, i.e., a highly defective DGEBA/Adenine molar ratio 29.4/6.0. Assuming that Adenine can contribute to the cross-linking reaction with at least the three amine functionalities (primary and secondary amines) and discarding the potential contribution of the aromatic ones, the epoxy/nucleophilic active sites ratio becomes 58.8/18, that is, about 30% defective with respect to the molar stoichiometric requirements. 

Notwithstanding such limitation, with respect to the commercial recipe and to the reaction stoichiometry, three different formulations were obtained starting from the highest attainable adenine/DGEBA molar ratio (Table 1) and they were tested to be evaluated as potential thermosets for further composites production; CLR/Adenine formulations are labelled CAx, according to Table 1. It is worth to note that the CLR resin producers (Entropy Resins), besides reporting the actual biobased content, state that their product is obtained from other processes’ byproducts and waste [53]; this observation, together with the attribution of the CLR ^1^H NMR spectrum, hints at the fact that the biobased component in the epoxy precursor is the glycidyl residue. Based on this assumption, not only the carbon-based content of the overall thermoset formulation has been evaluated, but also the overall biobased mass fraction (Table 1). Indeed, while adenine is fully biobased, the overall contribution to the resin’s carbon balance is limited, owing to the high presence of nitrogen in the ring structure, with a molecular formula C_5_H_5_N_5_, while it is more significant on the biobased mass balance. However, notwithstanding the limited amount of hardener used in the present formulations, adenine addition positively, even if not dramatically, impacts these indexes. 

### 3.1. Investigation of Curing of CLR—Adenine Formulations 

The number of available techniques able to explore the efficiency of the curing process in thermosetting systems is limited due to the lack of solubility of the forming cross-linked networks. In this work, the main aim of the investigation is a preliminary assessment to prove adenine ability to cross-link epoxy derivatives; hence, a fast and reliable approach is required. In this frame, a calorimetric investigation via DSC measurements of the different obtained formulations should be able to provide informative results. While this approach does not provide a full and conclusive scheme of the reactions mechanism, it nevertheless leads to obtaining some crucial information in a single measure for evaluating the reliability of the hardening system. Indeed, a DSC measurement is able to evidence the occurrence of the exothermic epoxy opening reaction via the detection of an exothermic peak, allowing one to also identify the typical temperature range required for such a reaction to proceed. Moreover, upon re-heating the same sample, it is also possible to obtain information on the glass transition temperature reached by the thermoset. The latter is a significant parameter for understanding the potential of the proposed hardener to positively cross-link the epoxy resin.

**Figure 1:**
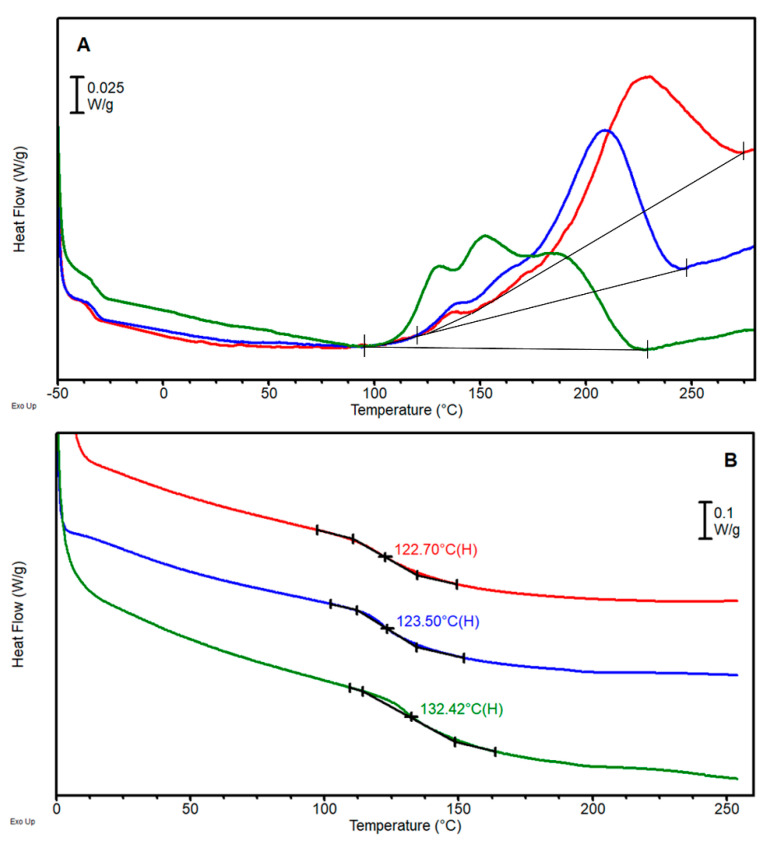
DSC first (**A**) and second (**B**) DSC heating scans of CA1(**—**), CA2 (**—**) and CA3 (**—**).

The thermal characterizations of the crude formulations CA1, CA2 and CA3 were thus carried out by DSC (Figure 1) at constant heating rate, with a first heating rate of 1 °C/min. This condition was chosen for its similarity to the autoclave heating rate and allows one to determine, in the first instance, the temperature range in which the reaction occurs, and the temperature of the maximum cross-linking rate (*T*_r max_), defined as the temperature of the most intense exotherm peak in the thermogram, is taken as an indication of the broader temperature range in which the reaction occurs (Table 2). Moreover, an attempt at determining the overall enthalpy of cross-linking has been conducted, as reported in Table 2. After a fast cooling, a further heating scan (20 °C/min) allows the determination of the *T*_g_ of the cross-linked material (Table 2) and the possible presence of any residual cross-linking, highlighted via detection of an exothermic signal at temperatures higher than the attained *T*_g_. 

As shown in Figure 1A and in Table 2, in the first heating scan, all the analyzed formulations display a similar low-T stepwise transition ascribed to the crude epoxy precursors glass transition, and at a higher temperature exothermic phenomenon, which can be associated to the cross-linking reaction between Adenine and the pre-polymer. For the latter event, a reaction enthalpy in the range 315–350 J/g, and increasing with the hardener fraction, was determined. However, it is worth pointing out that this growth is not proportional to the hardener fraction increase. Furthermore, it is possible to observe a shift towards lower temperatures (from 207 to 142 °C) of the *T*_r max_, depending on the amount of Adenine used. This behavior suggests that the cross-linking reaction occurs at lower temperature and with higher conversion by increasing the hardener quantity. It is worth noting that the exotherm displayed by the different formulations has a different shape and intensity based on the amounts of hardener added. In particular, both CA1 and CA2 show one main exothermic signal of different intensity with negligible shoulders at lower temperature, whereas in CA3 formulation, the exothermic transition is complex and multipeaked. This behavior suggests the occurrence of several sequential or simultaneous reactions curing cross-linking.

All the second heating scans (Figure 1B) show only a stepwise transition, positioned at significantly higher temperature than in the previous scan, and assigned to the resin *T*_g_: the actual value shifts towards higher temperature (123–132 °C), the greater the fraction of hardener. This behavior is in accordance with the registered increasing cross-linking reaction enthalpy by increasing adenine fraction and is related to the extent of the cross-linking that can occur in the system. While a greater quantity of hardener involves a higher number of reactive amino groups able to open the epoxy rings of the polymer precursor, the networks that set in also hamper a linear increase in the cross-linking that is proportional to the amount of hardener added. Finally, no exothermic transition can be detected, thus confirming the full conversion of the reacting moieties during the first heating step, and probably limited by the defective amount of adenine, owing to solubility limitations. 

The DSC results altogether demonstrate that the use of Adenine as a hardener for the epoxy system is effective and promising. Hence, with the aim of producing composites with different types of sustainable fibers, an attempt to evaluate the best performance in isothermal curing conditions was carried out. Thus, isothermal DSC scans of CA1, CA2, and CA3 formulations were carried out at 160, 180 and 200 °C. These temperatures were chosen on the basis of the position of the exothermal peaks location shown in the dynamic DSC analysis. Once the isotherm step at the selected temperature was completed, the samples were rapidly cooled and then heated up from 0 to 260 °C at 20 °C/min to determine the *T*_g_ of the obtained resin (Table 3) and to highlight the presence of any residual cross-linking. The obtained data highlight, as expected, a clear shift towards higher *T*_g_, increasing either the adenine content and/or the reaction temperature.

However, a twofold situation can be clearly observed: lower-T processes and low adenine content led to clearly poor *T*_g_ values and residual enthalpy in the second scans led to sign of uncomplete reaction, while a threshold *T*_g_ of about 145–150 °C can be obtained at higher processing temperature and higher adenine content (even if still highly defective with respect to stoichiometry). These results suggest that CA2 formulation could reach results comparable to CA3, already working at 180 °C. Indeed, processing the composites at 200 °C hot spots can, as previously reported, form activating parasitic reactions, which could lead to the loss of aromaticity due to the opening of the Adenine ring [54], with a consequent significant decrease in rigidity and in the system’s *T*_g_, as observed from the data in Table 2. Furthermore, it is worth noting that the formation of any hot spots can affect the structure of the fibers, especially the natural ones that are thermolabile, thus negatively influencing the thermal and mechanical performance of the final composite. At 180 °C, the reaction takes place more slowly than at 200 °C, with a lower tendency to hot-spot and allowing a better control over the exothermic cross-linking process. The latter temperature was thus selected for the composites curing process.

### 3.2. Study of Cross-Linking Reaction Mechanism

The understanding of the reaction mechanism leading to the final thermoset material structure can contribute towards reaching a better control of the cross-link formation, and in turn, of the properties of the final material. Unfortunately, the product of the cross-linking reaction between the epoxy resin precursor and hardener being an infusible and insoluble material with a highly branched/cross-linked structure, it is barely characterizable with common spectroscopic analysis such as NMR.

**Scheme 2:**
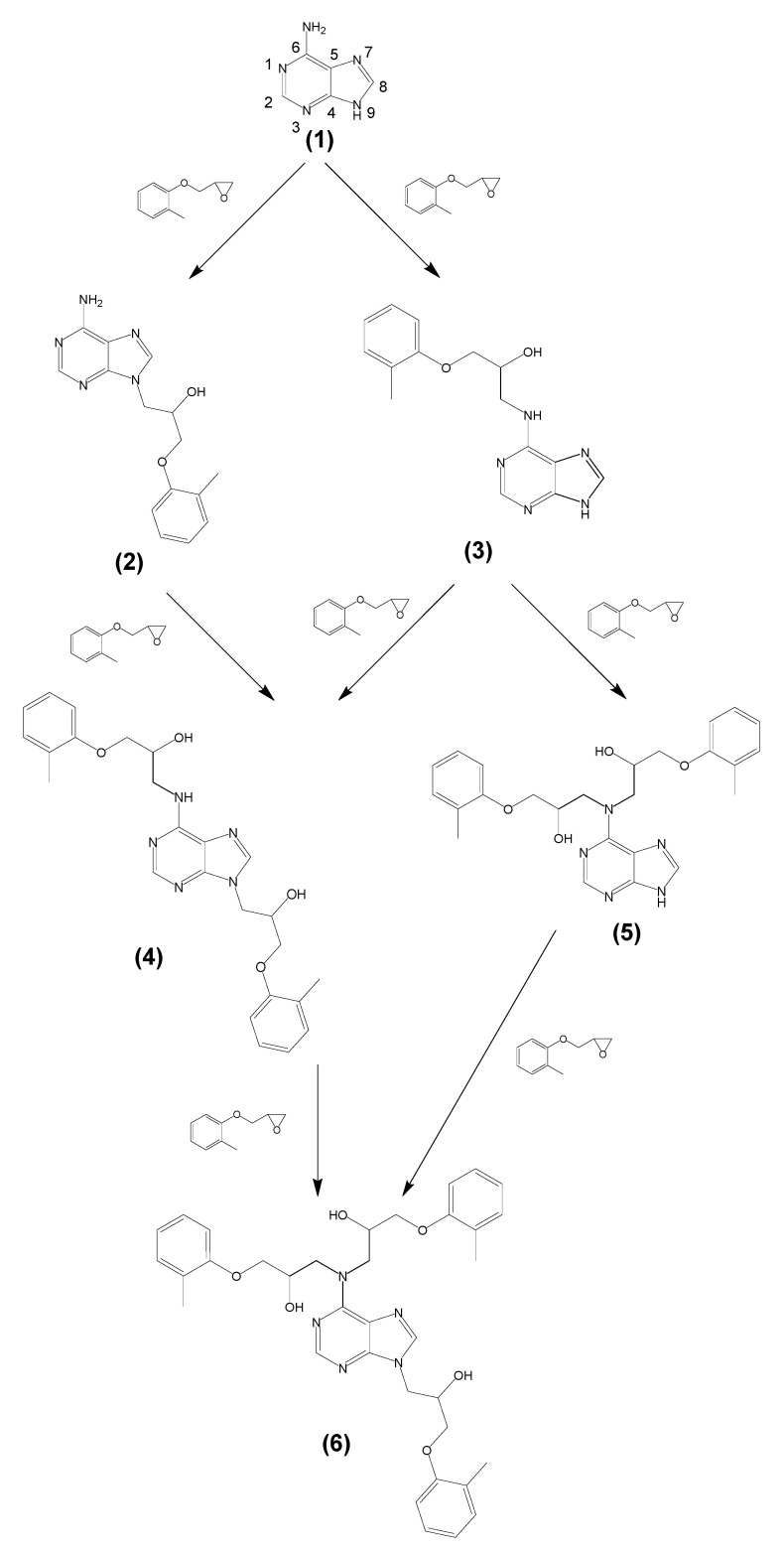
Possible reaction products between G2MPE and Adenine.

For this reason, Glycidyl 2-Methylphenyl Ether (G2MPE) was selected as a mono-epoxy model compound for the investigation of the reaction pathway with Adenine. G2MPE possesses a structure very similar to DGEBA (Scheme 2), but bearing only one epoxy functionality, allowing for a soluble reaction product. The cross-linking reaction was investigated through ^1^H NMR analysis.

Due to Adenine not being soluble in most common solvents, the reaction was carried out in DMSO, varying the molar ratio between the two reagents from equimolar conditions (G2MPE/Adenine 1:1) to an excess of epoxy compound (G2MPE/Adenine: 4:1 and 8:1). The experimental choice of working in the equimolar condition corresponds to an excess of amine moieties working with an epoxy/nucleophilic sites ratio 1/3 and it was addressed to attempt at discriminating the different reactivity of the amine residues toward a DGEBA-like epoxy functionality. The other chosen compositions, instead, refer to an excess of epoxy groups, that is more resembling of the actual cross-linking conditions carried out in the bulk. The obtained mixtures were named, respectively, GA1-0, GA4-0 and GA8-0. All the reactions were carried out in DMSO at 90 °C for 24 h. Scheme 2 shows the possible reaction products obtained by the reaction of the G2MPE epoxy ring with the primary and secondary amines of Adenine up to the three-substituted compound (Product 6). 

**Figure 2:**
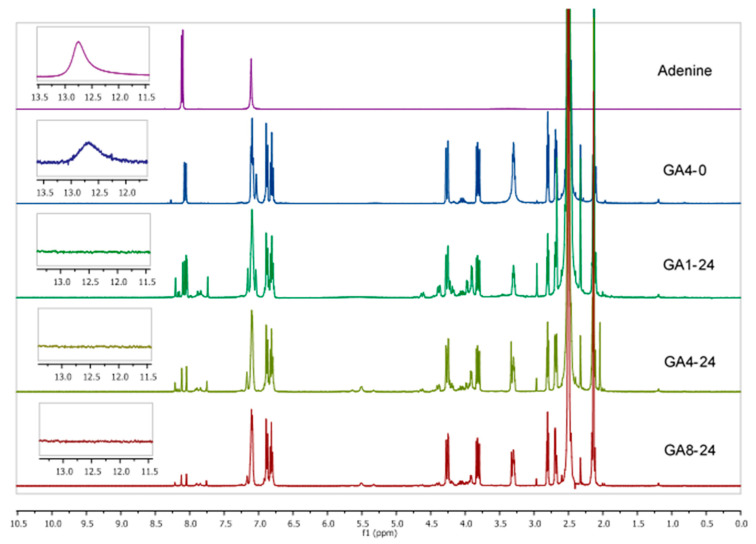
^1^H NMR spectra of Adenine, GA4-0 (**—**) and GA1-24 (**—**), GA4-24 (**—**), GA8-24 (**—**) after 24 h of reaction at 90 °C.

**Figure 3:**
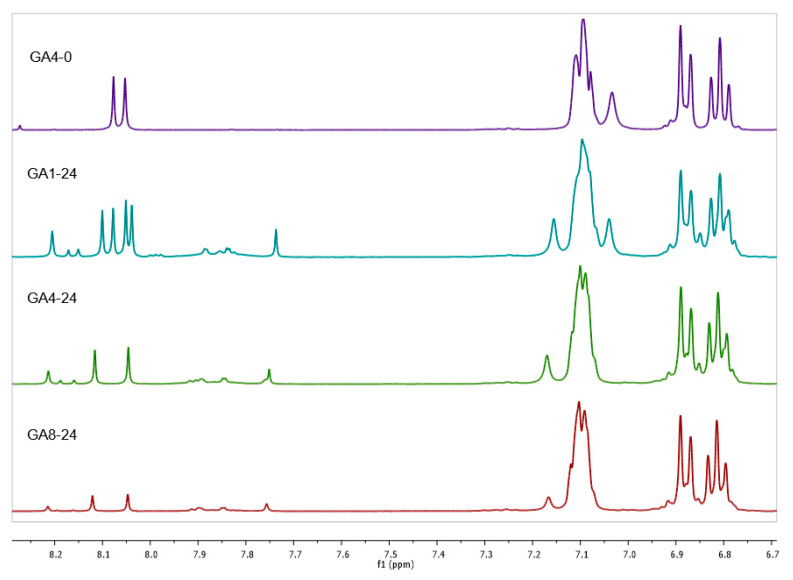
Magnification of ^1^H NMR spectra (aromatic range) of GA4-0 (**—**) and GA1-24 (**—**), GA4-24 (**—**), GA8-24 (**—**) after 24 h of reaction at 90°C.

The ^1^H NMR spectra of Adenine, G2MPE/Adenine 4:1 mol/mol mixture before reaction (GA4-0), and G2MPE/Adenine 1:1, 4:1 and 8:1 mol/mol after 24 h at 90 °C (named, respectively, GA1-24, GA4-24 and GA8-24) were recorded (see Figure 2 and Figure 3 for a focus on the most relevant spectral range). The spectrum of the mixture before the reaction (GA4-0) shows all the signals related to the two reactants. In particular, Adenine displays a signal at 12.8 ppm, which can be ascribed to the most acidic proton of the molecule; the one linked to the imidazole nitrogen (N(9) in Scheme 2); two signals around 8 ppm, corresponding to the two aromatic protons (C(2) and C(8) in Scheme 2); and another one at 7.0 ppm, due to the amino group in C(6). In addition, it is possible to observe the signals belonging to G2MPE, in particular, the ones of the aromatic protons around 7 ppm and the characteristic signals of the aliphatic CH of the epoxy ring between 3.5 and 2.5 ppm. After 24 h of reaction in equimolar conditions (GA1-24), the ^1^H-NMR spectrum shows the appearance of signals between 4.5 and 3.5 ppm, which can be ascribed to the formation of CH–OH and CH_2_–N groups, thus confirming the occurrence of the reaction between Adenine and the epoxy group. In addition, as shown in Figure 3, the two singlets of the Adenine aromatic protons (C(2) and C(8)), clearly visible around 8 ppm in GA4-0 spectrum, decrease in intensity and other two signals appear.

Furthermore, it is worth noting that after a 24 h reaction time, the signal at 12.8 ppm completely disappears (inset in Figure 2), whereas the signal at 7.1 ppm is still present (Figure 3). This behavior seems to suggest that the nucleophilic substitution reaction mainly involves the imidazole nitrogen atom (N(9)), thus leading to Product 2 in Scheme 2, as previously reported by Lubczak for the reaction between Adenine and ethylene oxide [55].

By magnifying the spectra (Figure 3), it is also possible to notice that the signal corresponding to the Adenine primary amine (at 7.05 ppm)—in the presence of equimolar amounts of the two reagents, after 24 h at 90 °C (GA1-24)—decreases in intensity and then completely disappears in excess of epoxy compound (G4-24 and G8-24). Simultaneously, a new signal at 7.18 ppm is formed. This behavior could be ascribed to the reaction of the amino group in C(6) with an epoxy group, with the consequent formation of a secondary amine (Product 4 in Scheme 2). This evidence suggests the occurrence of a competition between the Adenine imidazole nitrogen N(9) and the primary amine in C(6) in the nucleophilic attack to the epoxy ring. This behavior is in accordance with DSC analysis (Figure 1) which, showing complex and multipeak exotherm transitions, suggests the occurrence of sequential or simultaneous reactions during the cross-linking. 

In order to get a further insight into the mechanism, the reaction between Adenine and G2MPE with a 1:4 molar ratio (GA4), was monitored over time by ^1^H NMR analysis. In particular, the reaction was carried out at 90 °C, recording the ^1^H NMR spectra at different reaction times: after 1, 3, 5, 7 and 9 h, and thus, the correspondent samples were called GA4-1, GA4-3, GA4-5, GA4-7 and GA4-9, respectively. 

**Figure 4:**
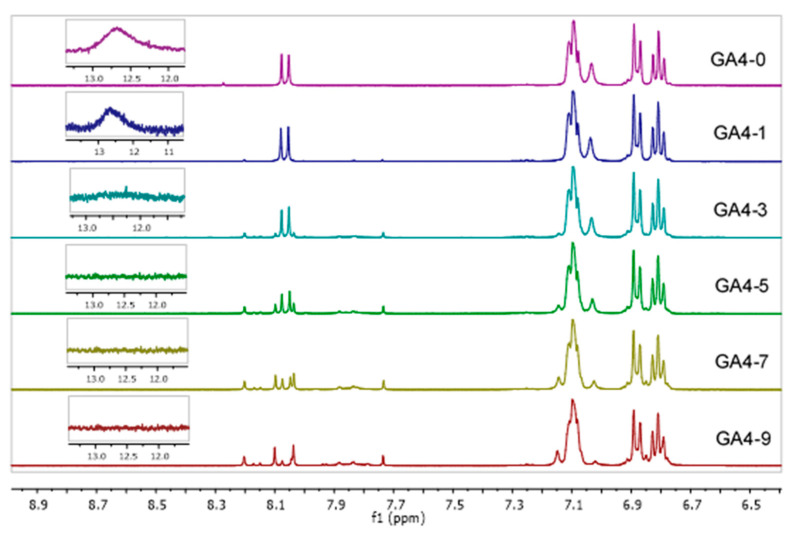
^1^H NMR spectra of GA4 at 0 (**—**), 1 (**—**), 3 (**—**), 5 (**—**), 7 (**—**), and 9 (**—**) h of reaction at 90 °C.

As reported in Figure 4, the signal at 12.8 ppm—characteristic of the imidazole nitrogen N(9) —decreases in intensity after 1 and 3 h, until disappearing at longer time; the signal at 7 ppm, corresponding to the NH_2_ protons, remains almost unchanged. This behavior suggests that G2MPE reacts with the secondary amine of the imidazole ring, thus leading—after three hours—to the complete substitution of the imidazole nitrogen N(9) and to the formation of Product 2 (Scheme 2). For longer reaction times, the signal of the primary amine at 7.05 ppm decreases in intensity and a new band at slightly lower fields (7.18 ppm) is observed. This behavior suggests that the primary amine of Adenine reacts with the epoxy compound, resulting in the formation of a secondary amine (Product 4, Scheme 2). Concerning the signal associated with the aromatic Adenine CH groups (at 8.1 ppm), after 3 h (G4-3), the NMR spectrum shows the formation of a multiplet, which suggests that some changes have occurred in the chemical surrounding and which resolves again in two singlets, at slightly different fields, after 9 h of reaction. 

The comparison of the integral of the signal of the unreacted epoxy CH_2_–O—with respect to those of CH_2_–O–Ar, CH_2_–N, and reacted epoxy CH–OH after 9 h at 90 °C—suggests a formation of the bi-substituted product (Product 4 in Scheme 2): the primary amine seems unable to completely react and form the three-substituted product (Product 6, Scheme 2), probably because of steric hinderance. The same issue can also be invoked for the aromatic nitrogen atoms’ involvement in the reaction. Similar results were obtained when raising the reaction temperature to 95 °C (as reported in Supplementary Materials, Figure S2).

**Scheme 3:**
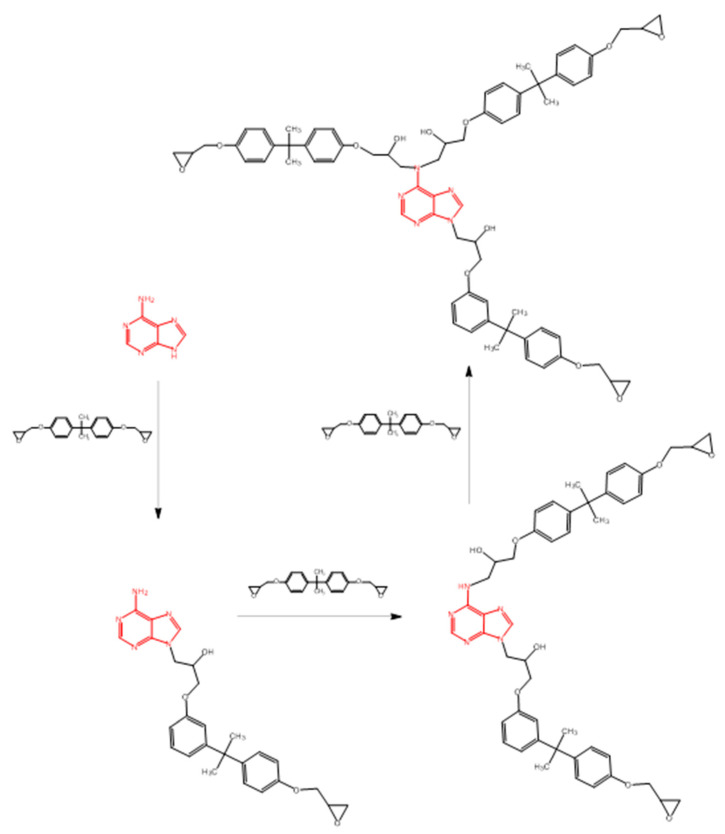
General synthetic reaction mechanism of Adenine with epoxy derivatives.

It is clear that, in the mild conditions (temperature below 100 °C) presently applied, it was not possible to push the reaction to the formation of the three-substituted compound. Despite this, increasing the temperature up to the real conditions used in the real curing processes of epoxy resins, the subsequent reaction of the secondary amine with formation of the desired product (Product 6), is reasonably attainable. This study allows, nonetheless, the determination, for the first time, of the sequence of consecutive reactions that leads to the formation of the three-dimensional lattice of a generic epoxy resin in the presence of Adenine. In particular, it was confirmed that the reactivity order of functional groups in Adenine is N(9) > NH_2_ in C(6) [55]. Thus, the reaction pathway of the cross-linking reaction between Adenine and a generic epoxy resin precursor could be envisaged, imagined as represented in Scheme 3.

### 3.3. Production and Characterization of CLR—Adenine (CA) Composite Materials with Natural Fibers and Recyled Carbon Fibers

Taking into account the promising results reported above, and in order to verify the application of the selected formulation, CA2 was employed as a thermosetting matrix to produce short fiber reinforced composites (SFRCs). Although SFRCs usually exhibit lower mechanical properties than long fiber counterparts, they can nonetheless be applied in a variety of fields, as they exhibit lightness and isotropic properties and allow a simpler manufacturing process than for long fiber composites [56,57,58,59,60]. Moreover, it must be considered that when recycled carbon fibers (rCFs) are specifically used for composite production, there is no preliminary control over their shape and size: they are indeed a secondary raw material, which, as previously reported, can maintain mechanical properties up to about 80%/90% of the virgin ones, depending on the applied recycling process [30,43,61,62,63]. In the case of rCFs, SFRCs—analogue composites with virgin carbon fibers (vCFs)—were also produced, for the sake of comparison, using in both cases a 45:55 matrix fiber ratio. Furthermore, with the aim to produce new sustainable composites, the bio-based resin/adenine system was also reinforced with natural short fibers, such as flax (FF) and jute (JF), using a 50:50 matrix/fiber ratio. Natural fibers represent a potential starting point for the design and production of new sustainable composite materials, obtained entirely from renewable sources. The slightly higher matrix content used with natural fibers is due to the difficultly of handling of the non-industrial mixing step of NF with the crude matrix.

All the composite materials were produced using a steel mold specially manufactured for this task which allows the excess of resin to bleed out prior to reaching gel point during the initial steps of the curing process that are carried out in a hot-press. In this process, composites—obtained as flat panels—were weighted in order to determine the actual fiber/resin ratio: indeed, assuming that the fraction of fiber cannot flow out the mold, and consequently the weight of the reinforcement remains constant, this assessment provides a rough estimation of the actual fiber content in the obtained composites. In particular, CF content was found to be about 75%, whereas the average natural fiber content was slightly lower, in the 60–70 wt% range (Table 4).

**Figure 5:**
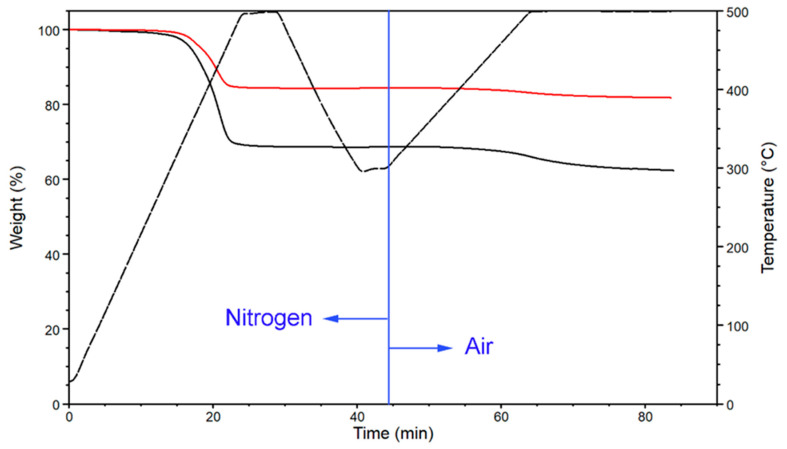
TGA thermograms of CA-vCF (**—**) and CA-rCF (—) composites. The dashed line represents the temperature program during the measurement, while the blue line shows the switch from nitrogen to air atmosphere. Curves displayed in the figure were selected to best fit the average values calculated for each batch of specimens.

In order to estimate the homogeneity of the fiber content, as well as to evaluate its thermal stability, all the composite materials were tested via TGA analysis. In the case of carbon fiber reinforced composites, a previously reported TGA program was applied [30] that allows for determination of the actual local carbon fiber content. This method, sketched with the dashed line in Figure 5, is based on a first degradation step carried out up to limited temperature (500 °C) in inert atmosphere to pyrolyze the matrix without significantly affecting carbon fibers; then, after cooling the sample down to 300 °C and switching to oxidizing atmosphere (air), a second heat treatment—once again up to 500 °C—was applied. In this way, the carbonaceous residue from pyrolytic degradation of the resin was removed, once again preserving the reinforcement, thus leading to a reliable estimate of the carbon/fiber ratio. Since, in this case, the sample dimension was extremely limited (only a few mg sized specimens can be tested in TGA), the measurement was repeated at least three times in different regions of the panel: in this way, not only an average evaluation of the fiber composition is provided, but it is also possible to estimate the homogeneity of the produced samples (Table 5). The thermograms obtained (Figure 5) for the CF-based composites show that the panel containing vCF display a solid residue, ascribed to the fibrous fraction. The average obtained value of such residue (Table 5) compares well with the gravimetric evaluation of fibers content described previously (Table 4), in the case of CA-vCF, while in the case of CA-rCF, the obtained value significantly differs from the gravimetric evaluation.

This fact, together with the higher standard deviation, accounts for a more inhomogeneous system when using recycled carbon fibers, and this can be the result of both the manual mixing step, as well as the lack of sizing in the recycled fibers, which somehow might hamper a homogenous mixing with the resin. While recycled fibers proved a good affinity with commercial epoxy resin formulation, it can be hypothesized that the use of adenine as a hardener might change the resin formulation affinity with the unsized, slightly surface oxidized CFs [64]. Both composites, however, display a good thermal stability, with an initial degradation temperature above 350 °C in both samples.

**Figure 6:**
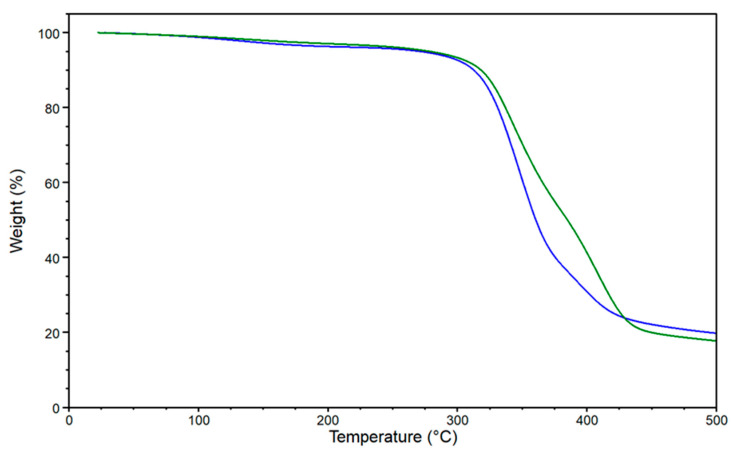
TGA thermograms of CA-FF (**—**) and CA-JF (**—**) composites. Curves displayed in the figure were selected to best fit the average values calculated for each batch of specimens.

Composites reinforced with natural fibers show a completely different behavior when subjected to TGA testing (Figure 6). Both CA-FF and CA-JF display an initial weight loss (Table 5), absent in the CF-based sample, at temperatures that are too low to be ascribed to thermal degradation (*T* < 110 °C): this phenomenon might be related, instead, to the extremely high hydrophilicity that characterizes cellulosic fibers [27], with high equilibrium moisture content. While fibers were dried before use, their handling in atmospheric humidity makes the fibers take up water even during their processing. Moreover, the cut of the panel, exposing natural fibers unprotected by the resin, favors once again water uptake. As discussed in the introduction, this is one particularly critical point in natural fiber composites production and application. Even when discarding such an initial weight loss, natural fiber composites display an overall lower thermal stability by almost 50 °C (Table 5), possibly due to the lower cellulosic fiber thermal stability [65]. Finally, both composites display a complex weight loss pattern that even in inert conditions leads to a residue well below 20% of the initial weight. This behavior implies that during the heating step in nitrogen atmosphere natural fibers undergo thermal degradation. Indeed, these fibers are prone to thermally degrade, even in the lack of an oxidative environment, making the TGA test not suitable for estimating the reinforcement fraction, since there is no way to separate the contribution to the weight loss of the matrix thermal decomposition from the one due to the fiber’s degradation. Hence, in this case, the only reliable for actual fiber/matrix composition value is the one stemming from gravimetric measuring of the panels (Table 4).

Each composite panel was cut in coupons suitable for further characterization (Figure S2, Supplementary Materials), each of which was measured and weighed in order to obtain an index of the compactness of the sample, defined as apparent density (AD). The AD of each coupon was then averaged in order to provide information about the overall homogeneity of the produced composites. As reported in Table 4, the produced carbon fiber composites possess average apparent densities (AAD) ranging from 1.45 to 1.49 g/cm^3^, in agreement with the expected density for a quasi-isotropic epoxy CFRC (1.55 g/cm^3^) [57]. It is worth noting that, in this case, CA-rCF has an average higher AAD, beside TGA having displayed some more inhomogeneous fiber/matrix distribution. This behavior can be ascribed to the lack of sizing, which indeed tends to maintain fibers still well-organized in bundles after cutting to the appropriate length, thus hampering the optimal compaction [66]. Both natural fiber reinforced composites display lower density values with respect to CF-based analogues, in the range 1.15 g/cm^3^ (CA-FF) and 1.03 g/cm^3^ (CA-JF). Such a behavior is expected, and indeed observed, since the density of natural fibers is significantly lower than carbon ones [29]. Thus, the determined AADs suggest that a good compaction has been reached for all the produced composites, although a manual lab scale process was used, and in presence of a high final fiber dosage (about 60–80%).

Finally, dynamic mechanical analysis (DMA) was carried out with the aim of providing comparative properties of the composites obtained with recycled fibers with respect to those obtained from virgin pristine fibers as well with those obtained from natural fibers. The results reported in Table 4 show that higher mechanical performances were obtained, as expected, for carbon fiber reinforced materials with respect to natural fibers. Adenine is thus able to promote an efficient and reliable cross-linking for epoxy-based precursors, leading to composites with excellent mechanical properties. In particular, the highest value of elastic module E’ was obtained for composites based on virgin carbon fibers (CA-vCF) and once again the presence of the sizing treatment can be invoked for having promoted an optimal fiber/matrix adhesion, besides the fibers probably performing slightly better from a mechanical point of view. However, the recycled fibers were proved to behave quite closely to the pristine ones. Such an observation is highly promising, since the gap in mechanical performance might be bridged by application of a convenient surface treatment of the recycled CF tailored for adenine-based resins formulations. Despite this, the obtained results can be considered highly satisfactory for the application of recycled fibers as reinforcing agents, having produced short fiber composites with high moduli. Materials reinforced with natural fibers show also a promising elastic modulus, although, as expected, this is lower than for carbon fibers. The best results have been obtained with jute fibers, whose composites show a higher modulus than the flax-based analogues.

**Figure 7:**
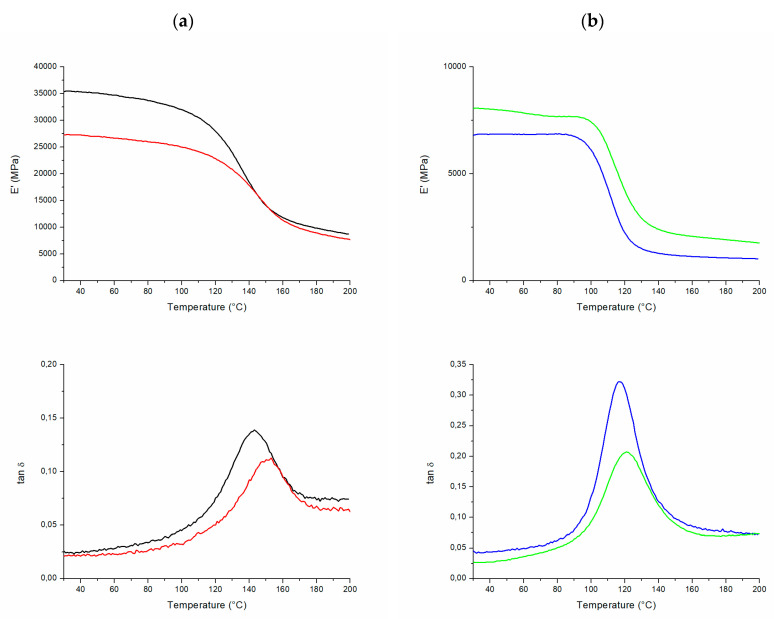
DMA spectra of (**a**) carbon fibers (CA-vCF (**—**) and CA-rCF (**—**)), and (**b**) natural fibers (CA-FF (**—**) and CA2-J (**—**))-based composites samples.

Analyzing the E’ onset temperatures (E’ onset) of the composites (Figure 7 and Table 4), that is taken as an indication of the glass transition in the most conservative approach from a mechanical point of view, the samples with virgin and recycled carbon fibers show a similar behavior, with thermal stability that is excellent. In the same frame, the results obtained with flax fibers are similar to those obtained with jute fibers and are remarkable, when taking into account the problems that natural fibers might undergo in epoxy resin cross-linking, owing to the water uptake that could interfere with the curing.

The tan δ curves have been also reported in Figure 7b, since evaluation of tanδ maximum is probably the most common approach to define the glass transition of the composite materials (Table 4). All the analyzed composites show a single relaxation process (*T*α) that can be associated with the glass transition of the resin (*T*_g_), whose position depends on the type of fiber/resin system. Once again, the best results were obtained when virgin and recycled carbon fibers were used. The two composites derived from carbon fibers show a tan δ peak of 144 °C (CA-vCF) and 147 °C (CA-rCF), values that are compliant with high-T (HT) performing materials, while products with natural fibers show a tan δ peak as high as 115–120 °C (Table 4), which are excellent results for commonly applied composites.

In conclusion, the overall obtained results confirm a good performance of the adenine-based resins and a good interaction between all types of fibers and CA system; furthermore, the possibility of producing composite materials with good *T*_g_ and high mechanical properties is confirmed, supporting the use of Adenine as an efficient cross-linking agent for epoxies.

## 4. Conclusions

In the present work, a green resin-hardener system based on adenine and an epoxy partially biobased precursor was studied and its cross-linking ability was investigated. Different formulations up to 35% biobased mass content were achieved and they all proved to be able to positively cross-link with good results. An investigation of the potential performance obtained in isothermal condition suggested that 5% hardener was able to provide good thermal performance and this formulation was thus used to produce composites, although a high T curing process is required (at least 160 °C). In composites production, recycled carbon fibers, as well as jute and flax natural fibers, were used as sustainable reinforcements. Virgin carbon fibers were also used for the sake of comparison. The curing cycle was optimized for both carbon and natural fiber reinforced materials, with the aim to achieve the better final properties.

Composites display all good thermal and mechanical properties with glass transition in the range of HT resins (*T*_g_ < 150 °C) for the carbon fiber-based composites, which also display a good compaction and excellent moduli. The natural fiber-based composites display slightly lower performance that is nonetheless good comparing with standard composite performance. The present results thus pave the way to the application of adenine as hardener system for composites production. While the high temperature curing required to process the composites might appear to be a drawback of the system, adenine proved to represent a viable alternative as a hardener from renewable sources for epoxy resins in the future, since it leads to resins able to reach significant thermo-mechanical performance.

## Figures and Tables

**Table 1:** Composition of the different adenine-based formulation and an evaluation of their biobased content.

Sample	CLR (w%)	Adenine (w%)	CLR (g)	Adenine (g)	CLR (mmol)	Adenine (mmol)	Biobased C Content (%) ^1^	Biobased Mass Fraction (%) ^2^
**CA1**	97.7	2.3	10	0.235	29.4	1.7	29.2	34.1
**CA2**	95.5	4.5	10	0.471	29.4	3.5	29.8	34.8
**CA3**	92.5	7.5	10	0.810	29.4	6.0	30.3	35.6

^1^ The fraction of biobased carbon in the thermoset has been evaluated considering the commercial epoxy precursor biobased carbon content according to ASTM D6866 equal to 28.6% and the adenine biobased carbon content that is 100%, in the actual formulation composition; ^2^ The fraction of biobased mass has been evaluated considering the commercial epoxy precursor biobased mass content equal to 33.5% and the adenine biobased content that is 100%, in the actual formulation composition.

**Table 2:** Dynamic scanning calorimetry (DSC) data of CLR pre-polymer/Adenine (CA) formulations.

Sample	ΔH (J/g) ^1^	*T*_r max_ (°C) ^2^	*T*_g_ (°C) ^3^
**CA1**	316	217	123
**CA2**	325	207	124
**CA3**	350	142	132

^1^ ΔH is the heat generated by the cross-linking reaction; ^2^ *T*_r max_ is the temperature corresponding to the maximum rate of the cross-linking reaction; ^3^ Determined in second heating scan.

**Table 3:** Relevant isothermal DSC data of CA1, CA2 and CA3 at 160, 180 and 200 °C.

Sample	*T*_g_ (°C)	*T*_g_ (°C)	*T*_g_ (°C)
	*T*_iso_ 160 °C ^1^	*T*_iso_ 180 °C ^1^	*T*_iso_ 200 °C ^1^
**CA1**	/	68	117
**CA2**	57	152	142
**CA3**	168	147	147

^1^*T*_iso_ is the temperature chosen for the curing process in isothermal condition.

**Table 4:** Thermo-mechanical properties of different SFRCs based on Adenine-CLR matrix.

Sample	Fiber Fraction ^1^ (w%)	ADD ^2^ (g/cm^3^)	E’ ^3^ (GPa)	E’ onset ^3^ (°C)	tan δ peak ^3^ (°C)
**CA-vCF**	77	1.45	33.8 ± 1.6	119	144
**CA-rCF**	75	1.49	25.9 ± 1.5	120	147
**CA-FF**	70	1.15	7.2 ± 1.5	95	116
**CA-JF**	62	1.03	8.7 ± 2.1	101	120

^1^ Calculated from panels weighing after resin loss during curing; ^2^ average apparent density (AAD) determined as the average of the ratio between measured weight and volume of the single bars as cut in the convenient dimension for mechanical tests; ^3^ determined by DMA.

**Table 5:** TGA results of CA-based composites reinforced with either carbon fibers or natural fibers.

Sample	Weight Loss *T* < 110 °C (w%)	*T*_d onset_ (°C)	Residue (w%)
**CA-vCF**	/	359 ± 2	77 ± 2
**CA-rCF**	/	356 ± 2	69 ± 7
**CA-FF**	3 ± 1	316 ± 1	1 ± 1
**CA-JF**	3 ± 1	318 ± 2	1 ± 1

## Supplementary Materials

The following are available online at https://www.mdpi.com/2073-4360/12/12/3054/s1, Figure S1: ^1^H and ^13^C NMR spectra in CDCl_3_ of CLR resin with the signals attribution. Figure S2: ^1^H NMR spectra of Adenine, GA4-0, GA1-24, GA4-24, GA8-24 after 24 h of reaction at 95 °C. Figure S3: Pictures of the different composites obtained.
