# Supplementary material for: Adenine as Epoxy Resin Hardener for Sustainable Composites Production with Recycled Carbon Fibers and Cellulosic Fibers
Source: Polymers (Basel). 2020 Dec 20;12(12):3054. doi: 10.3390/polym12123054 (PMC7766952; doi:10.3390/polym12123054)
Supplement: Supplementary file 1 [file polymers-12-03054-s001.pdf]

Adenine as epoxy resin hardener for sustainable composites production with recycled carbon fibers and cellulosic fibers

Stefano Merighi [1], Laura Mazzocchetti [1,2], Tiziana Benelli [1,2] and Loris Giorgini [1,2,*]

[1] Department of Industrial Chemistry "Toso Montanari"and INSTM UdR-Bologna, University of Bologna, Viale Risorgimento 4, 40136 Bologna, Italy;
[2] Interdepartmental Center for Industrial Research on Advanced Applications in Mechanical Engineering and Materials Technology, CIRI-MAM, University of Bologna, Viale Risorgimento 2, 40136 Bologna, Italy;
* Correspondence: loris.giorgini@unibo.it; Tel.: +39-051 2093688

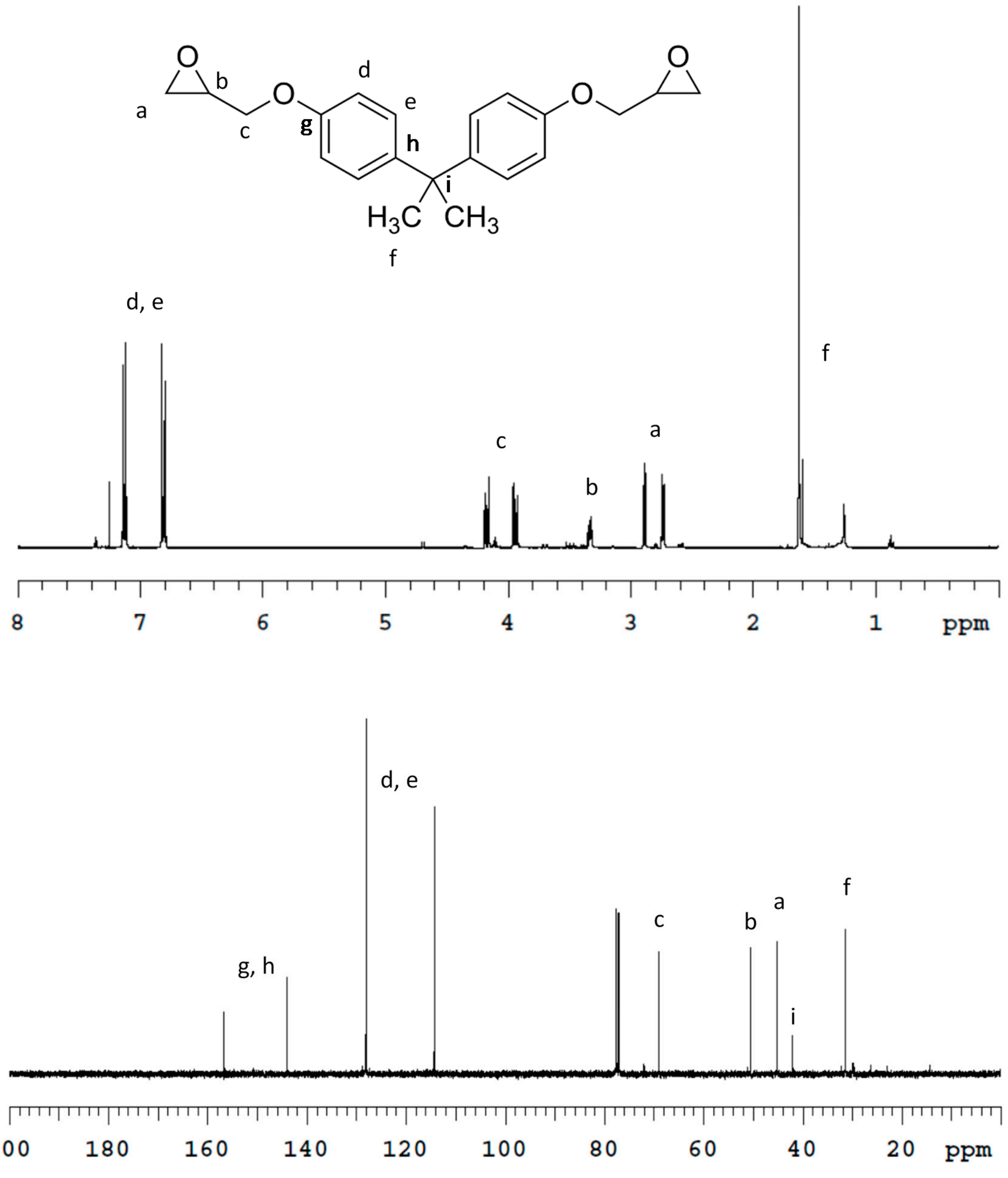

Figure S1. ^{1}H- and ^{13}C-NMR spectra in $CDCl_3$ of CLR resin with the signals attribution.

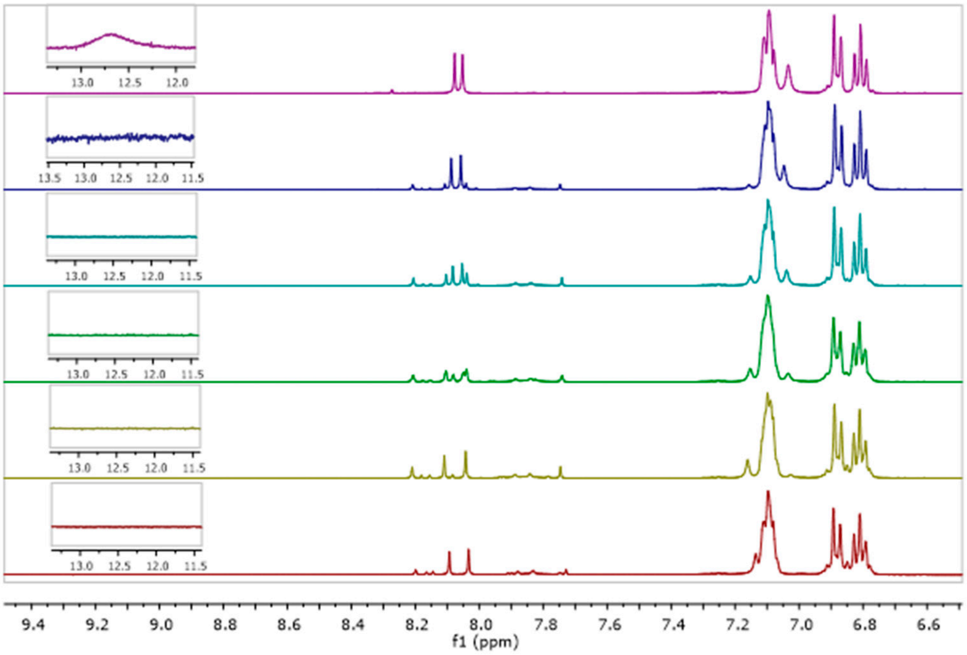

Figure S2. ^{1}H-NMR of GA4 system at 0 (—), 1 (—), 3 (—), 5 (—), 7 (—) and 9 (—) hours of reaction at 95°C.

In order to increase the reaction speed and push it until the possible formation of the three-substituted product, the reaction was carried out at higher temperature.

As reported in Figure S2, the NMR analysis highlights the disappearance of the signal at 12.8 ppm already after 1 hour at 95°C, thus suggesting the complete reaction of the imidazole nitrogen N(9). At the same time the signal corresponding to the primary amine in C(6) decreases, the signal associated to the aromatic CHs (at 8.1 ppm) shift and the formation of a multiplet is observed. By increasing the reaction time, an evident decrease in intensity of the signal at 7.05 ppm is observed, until its total disappearance, and a new band at 7.18 ppm is formed. These events are again associated with the partial substitution of NH_2, which forms a secondary pyrimidine amine. After nine hours of reaction, a different chemical shift of the signals related to the aromatic Adenine CHs is observed and it is possible to hypothesize the formation of the bi-substituted product (Product 4, Scheme 2). Even in these conditions, the NMR analysis does not show the formation of the three-substituted product (Product 6, Scheme 2), probably due to the steric hindrance present on the molecule.

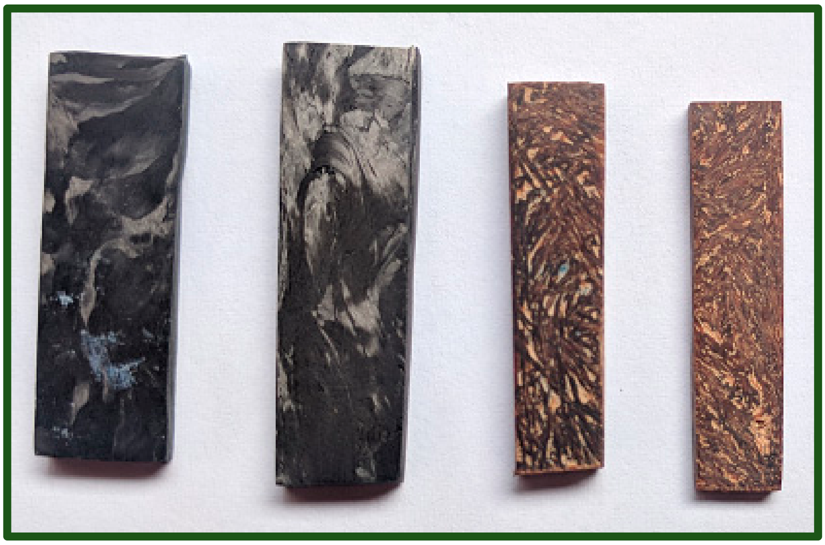

Figure S3. Pictures of the different composites obtained.
